# Transcriptional Regulation of an Evolutionary Conserved Intergenic Region of CDT2-INTS7

**DOI:** 10.1371/journal.pone.0001484

**Published:** 2008-01-23

**Authors:** Hiroki Nakagawa, Moe Tategu, Rieko Yamauchi, Kaori Sasaki, Sota Sekimachi, Kenichi Yoshida

**Affiliations:** Department of Life Sciences, Meiji University School of Agriculture, Kawasaki, Kanagawa, Japan; National Cancer Institute at Frederick, United States of America

## Abstract

**Background:**

In the mammalian genome, a substantial number of gene pairs (approximately 10%) are arranged head-to-head on opposite strands within 1,000 base pairs, and separated by a bidirectional promoter(s) that generally drives the co-expression of both genes and results in functional coupling. The significance of unique genomic configuration remains elusive.

**Methodology/Principal Findings:**

Here we report on the identification of an intergenic region of non-homologous genes, CDT2, a regulator of DNA replication, and an integrator complex subunit 7 (INTS7), an interactor of the largest subunit of RNA polymerase II. The CDT2-INTS7 intergenic region is 246 and 245 base pairs long in human and mouse respectively and is evolutionary well-conserved among several mammalian species. By measuring the luciferase activity in A549 cells, the intergenic human sequence was shown to be able to drive the reporter gene expression in either direction and notably, among transcription factors E2F, E2F1∼E2F4, but not E2F5 and E2F6, this sequence clearly up-regulated the reporter gene expression exclusively in the direction of the CDT2 gene. In contrast, B-Myb, c-Myb, and p53 down-regulated the reporter gene expression in the transcriptional direction of the INTS7 gene. Overexpression of E2F1 by adenoviral-mediated gene transfer resulted in an increased CDT2, but not INTS7, mRNA level. Real-time polymerase transcription (RT-PCR) analyses of the expression pattern for CDT2 and INTS7 mRNA in human adult and fetal tissues and cell lines revealed that transcription of these two genes are asymmetrically regulated. Moreover, the abundance of mRNA between mouse and rat tissues was similar, but these patterns were quite different from the results obtained from human tissues.

**Conclusions/Significance:**

These findings add a unique example and help to understand the mechanistic insights into the regulation of gene expression through an evolutionary conserved intergenic region of the mammalian genome.

## Introduction

CDT2, a WD40 domain-containing protein, was first shown to have an important role in DNA replication in both the mitosis and the meiosis of fission yeast [1]. Similar to fission yeast, the mammalian homologue of *Drosophila* CDT2 was found to associate with the Cullin-4 (Cul4) ubiquitin ligase containing the Cul4 scaffold and DDB1 adaptor protein [2]. In *Xenopus*, CDT2 is recruited to the replication forks via CDT1 and PCNA, where CDT1 ubiquitation occurs [3]. Loss of human CDT2 is known to result in suppression of CDT1 proteolysis in response to DNA damage and causes rereplication and checkpoint activation [3], [4]. CDT2 and PCNA were found to interact physically with a p53 tumor suppressor and its regulator MDM2, and those associations are involved in CDT1 degradation after DNA damage [5]. These reports strongly suggest that CDT2 is a conserved component of the Cul4-DDB1 E3 that is essential to destroy CDT1 and ensure proper cell cycle regulation and timing of DNA replication.

Even before the beginning of the planning of this study, we have been interested in the transcriptional regulation of CDT2 by the E2F transcription factors. Because CDT2 is tightly involved in the initiation of DNA replication and cell cycle regulation, and probably deregulated CDT2 expression could promote carcinogenesis accompanying genomic instability, these considerations strongly support the possibility that CDT2 could be a plausible transcriptional target of E2Fs. During the characterization of the putative promoter region of human CDT2, we noticed that the integrator complex subunit 7 (INTS7) gene is arranged in a head-to-head orientation to the CDT2 gene and separated by a small intergenic sequence. INTS7 has been identified as one of twelve novel subunits, which directly interacted with the C-terminal domain of the RNA polymerase II largest subunit, and was shown to be evolutionarily conserved in metazoans [6]. Recent genome-wide analyses of the mammalian genome revealed that many genes tend to be located in the close vicinity of, and approximately 10% of genes constitute neighboring paired genes to each other; arranged in an adjacent head-to-head orientation resulted in the sharing of regulatory sequence elements [7], [8].

The unique genomic configuration of CDT2 and INTS7, though presumed as having a different genomic function, prompted us to investigate whether the CDT2-INTS7 intergenic region could act as a bidirectional promoter, capable of coordinating the expression patterns of the two genes. In the present study we investigated whether the region shared by the two genes could possibly regulate bi-directional transcription, and that E2Fs could play a critical role solely in the regulation of CDT2. In addition, we planned to elucidate if the tissue and developmental expression patterns differed between CDT2 and INTS7, and also differed between human and rodent. If proved, our data might thus provide the first evidence that two genes, involved in DNA replication and transcription, respectively, could share a bidirectional promoter but have different regulatory mechanisms.

## Results

### *In silico* and molecular analyses of the mammalian CDT2-INTS7 intergenic region

**Figure 1:**
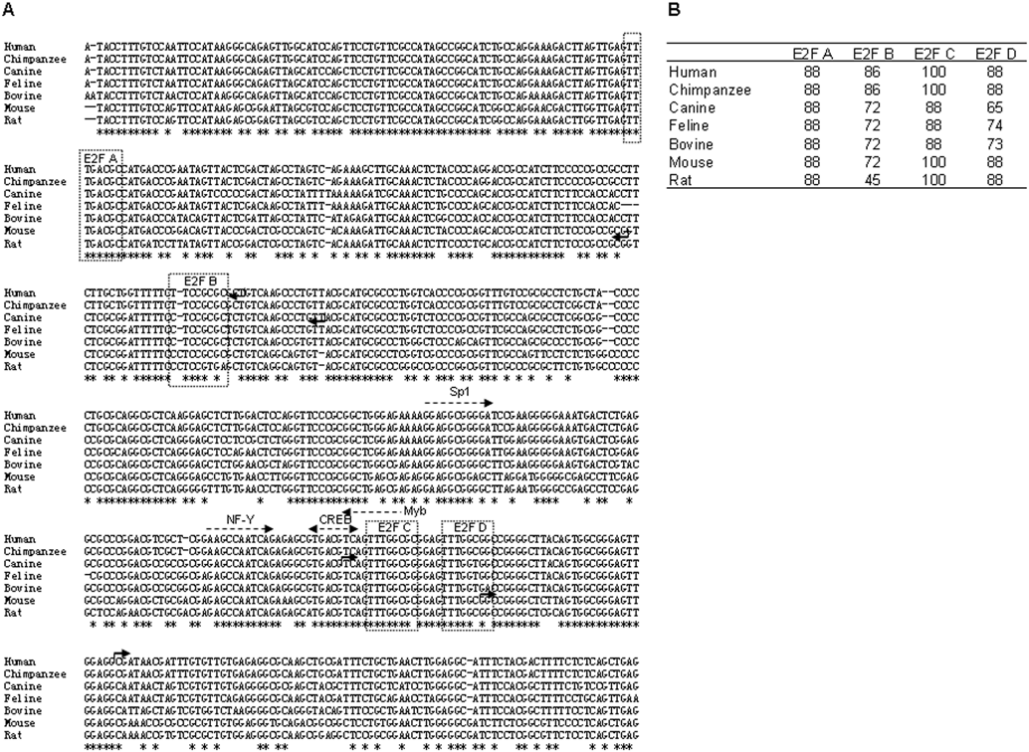
* In silico* analysis of the CDT2-INTS7 intergenic region. (A) Nucleotide sequence of the intergenic region of CDT2 and INTS7 genes. The sequences of seven mammalian genes are aligned and conserved nucleotides are marked with asterisks under the alignment. The bent arrows indicate the transcription start sites and direction of human genes (5′-CGATA— and 5′-AGCGC— for CDT2 and INTS7, respectively), canine genes, (5′-TCAGT— and 5′-AACAG— for CDT2 and INTS7, respectively), and mouse genes (5′-GGCGG— and 5′-CGCGG— for CDT2 and INTS7, respectively). The bent arrows positioned on the sequences are for CDT2. The bent arrows positioned under the sequences are for INTS7. Transfac software (threshold >80) predicts four E2F consensus sites (E2F A∼D, marked with dotted boxes), and Sp1 (5′-GAGGCGGGGA), NF-Y (5′-AAGCCAATCAG), CREB (5′-TGACGTCA), and Myb (5′-CCAAACTGAC) transcription factor-binding sites (marked by arrows with dotted lines). (B) Computer predicted threshold (Transfac software) of E2F A∼D were summarized for seven mammalian genes.

Sequence alignment of mammalian CDT2 and INTS7 intergenic region derived from human, chimpanzee, canine, feline, bovine, mouse, and rat sequences showed that the two genes had head-to-head orientation with high sequence identity (Fig. 1A, sequence identity marked by asterisks). The nucleotide length of the intergenic region was 246, 245, and 181 base pairs for human, mouse, and canine, respectively (Fig. 1A, bent arrows denote the transcriptional start sites of CDT2 and INTS7). *In silico* analyses with Transfac software (threshold >85) revealed that the region surrounding the CDT2-INTS7 intergenic region contained four putative E2F consensus sites (denoted E2F A∼D) (Fig. 1A, dotted boxes). In addition, Sp1 (threshold 91), NF-Y (threshold 90), CREB (threshold 100), and Myb (threshold 88) consensus sites were identified at the vicinity of the transcriptional start site of the CDT2 gene (Fig. 1A). Among four putative human E2F consensus sequences, E2F A and B were seen to locate downstream of the transcriptional start site of human INTS7, while E2F C and D clustered in proximity just upstream of the transcriptional start site of human CDT2 (Fig. 1A). Sequence comparison of four putative E2F consensus sequences among seven mammalian species revealed that E2F A and C were relatively well conserved during evolution, while E2F B and D were variable (Fig. 1B).

**Figure 2:**
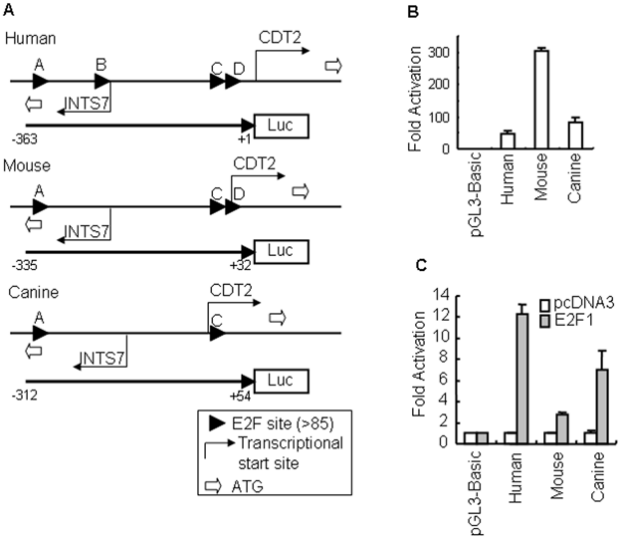
Promoter analysis of the CDT2-INTS7 intergenic region. (A) The structure of human, mouse, and canine CDT2 and INTS7 genes in the head-to-head orientation. Translation start codons (represented by ATG) of the CDT2 and INTS7 genes are marked by bold arrows in white. Transcription start sites are indicated by the bent arrows. The transcription start site of CDT2 is designated as “+1”. Positive (negative) numbers are assigned to nucleotides downstream (upstream) of nucleotide +1. Arrowheads indicate the E2F consensus sites (threshold >85). Arrows with numbers were the region and direction used for the luciferase (Luc) assay. (B) Luciferase expression of pGL3-human −363/+1, pGL3-mouse −335/+32, and pGL3-canine −312/+54 constructs in A549 cells are shown as fold induction with respect to the pGL3-Basic vector as 1. The values reported for transfection experiments are the means±standard deviation (n = 3). (C) Luciferase expression of pGL3-human −363/+1 (hereafter denoted as ABCD), pGL3-mouse −335/+32, and pGL3-canine −312/+54 constructs in A549 cells were up-regulated by co-expressing the E2F1, and are shown as fold induction with respect to the pcDNA3 vector as 1. The values reported for transfection experiments are the means±standard deviation (n = 3).

We first verified the promoter activity of the entire human, mouse, and canine intergenic regions, then we checked our hypothesis whether E2F1 could regulate CDT2 expression. For this purpose, we analyzed the promoter activity of the −363/+1, −335/+32, and −312/+54 intergenic region of human, mouse, and canine sequences, respectively (Fig. 2A, in which the transcription start site of CDT2 is designated as +1), by using firefly luciferase as the reporter gene. This region was PCR-amplified from genomic DNA. The fragment was then ligated in the CDT2 direction into the promoterless pGL3-Basic vector upstream of the luciferase coding region. Transient transfection assays in A549 cells revealed that the cloned genomic fragment was sufficient for the expression of the firefly luciferase gene. By comparison with the activity of the pGL3 vector alone normalized as 1, the human, mouse, and canine genomic fragments increased the luciferase activity by approximately 50-, 300-, and 100-fold, respectively, in the CDT2 direction (Fig. 2B). Co-expression of the E2F1 expression vector with the pGL3 vector series showed that human, mouse, and canine genomic fragments were responsible for ectopic E2F1 expression by approximately 12-, 3-, and 7-fold, respectively, compared to the activity obtained with pcDNA3 normalized as 1 (Fig. 2C). Taken together, these results indicate that the mammalian intergenic region could be a CDT2 promoter and is regulated by E2F1.

### Human CDT2-INTS7 intergenic region acts as a bidirectional promoter

**Figure 3:**
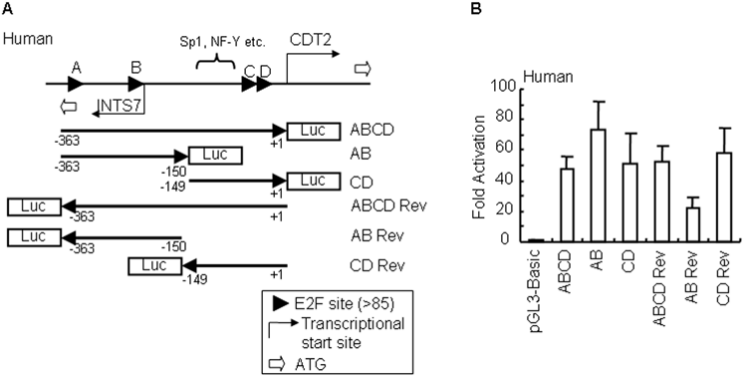
The human CDT2-INTS7 intergenic region acts as a bidirectional promoter. (A) Structure of human CDT2 and INTS7 genes in the head-to-head orientation. Translation start codons (represented by ATG) of CDT2 and INTS7 genes are marked by bold arrows in white. Transcription start sites are indicated by the bent arrows. The transcription start site of CDT2 is designated as “+1”. Positive (negative) numbers are assigned to nucleotides downstream (upstream) of nucleotide +1. Arrowheads indicate E2F consensus sites (threshold >85). Arrows with numbers represent the region and direction used for the luciferase (Luc) assay. (B) Luciferase expression of pGL3 constructs are summarized in (A) in A549 cells and are shown as fold induction with respect to the pGL3-Basic vector as 1. The values reported for transfection experiments are the means±standard deviation (n = 3).

The closeness of the transcription start sites suggested to us that the CDT2-INTS7 intergenic region could act as a bidirectional promoter. To check this notion, we cloned the human −363/+1 (hereafter denoted as ABCD because it contains E2F consensus sites E2F A∼D) region into a pGL3-Basic reporter plasmid in the reverse direction (designated as ABCD Rev). Further, we produced 5′ or 3′ half deleted constructs of ABCD (named AB and CD) and ABCD Rev (named AB Rev and CD Rev) (Fig. 3A). Deletion constructs were generated by cloning promoter PCR fragments in both directions upstream of the firefly luciferase gene into the pGL3-Basic vector. All constructs were checked for their promoter activity in transient transfection experiments into A549 cells. Promoter activity of the deletion fragments in either the CDT2 or the INTS7 direction was compared to that of the constructs containing the entire bidirectional promoter pGL3-ABCD and pGL3-ABCD Rev. Promoter activity was demonstrated almost equally except for AB Rev (Fig. 3B). But the activity of AB Rev (about 20-fold) was not so low when compared with the activities of other constructs (around 50-fold). Taken together, our results indicate that the CDT2-INTS7 intergenic region acts as a bidirectional promoter regardless of its orientation, and the functional minimal region was seen to be most probably inseparable within the intergenic region.

### Deletion analyses of the human CDT2 promoter

**Figure 4:**
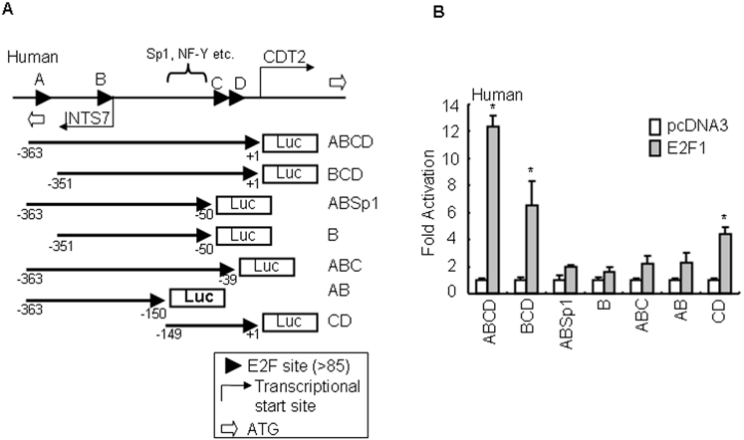
Deletion analyses of the human CDT2 promoter to identify the E2F responsive site. (A) Structure of the human CDT2 gene and location of a series of deleted constructs. Translation start codons (represented by ATG) of CDT2 and INTS7 genes are marked by bold arrows in white. Transcription start sites are indicated by the bent arrows. The transcription start site of CDT2 is designated as “+1”. Positive (negative) numbers are assigned to nucleotides downstream (upstream) of nucleotide +1. Arrowheads indicate E2F consensus sites (threshold >85). Arrows with numbers represent the region and direction used for the luciferase (Luc) assay. (B) The levels of luciferase expression of human CDT2 deleted promoter constructs in A549 cells were tested with E2F1 coexpression, and are shown as fold induction with respect to the pcDNA3 vector as 1. The values reported for transfection experiments are the means±standard deviation (n = 3; asterisk, *P*<0.05 for pcDNA3 versus E2F1).

To check which region of the human CDT2 promoter was responsible for E2F1-mediated CDT2 transcription, we made partial deletion constructs and checked for their promoter activity in transient transfection experiments (Fig. 4A). From the reporter assay conducted by the same protocol described above, we were able to note that pGL3-BCD (E2F A-deleted) decreased the promoter activity to a level one-half that of pGL3-ABCD, indicating that E2F B∼D could still stand as E2F regulatory elements and E2F A partially contributed to the E2F-mediated transcription (Fig. 4B). Deletion of E2F C/D (pGL3-ABSp1, pGL3-AB), E2F A/C/D (pGL3-B) or E2F D (pGL3-ABC) dramatically abolished the E2F1-induced promoter activity, suggesting the E2F D site or surrounding sequences could play a critical role in E2F1-mediated CDT2 expression (Fig. 4B). This is supported by the fact that pGL3-CD, in which E2F A/D is deleted, still exerted 5-fold promoter activity. These results suggested that E2F consensus sites adjacent to the transcriptional start site of CDT2 were most important for E2F1-mediated transcriptional regulation of CDT2.

### The human CDT2-INTS7 intergenic region is asymmetrically regulated by transcription factors

**Figure 5:**
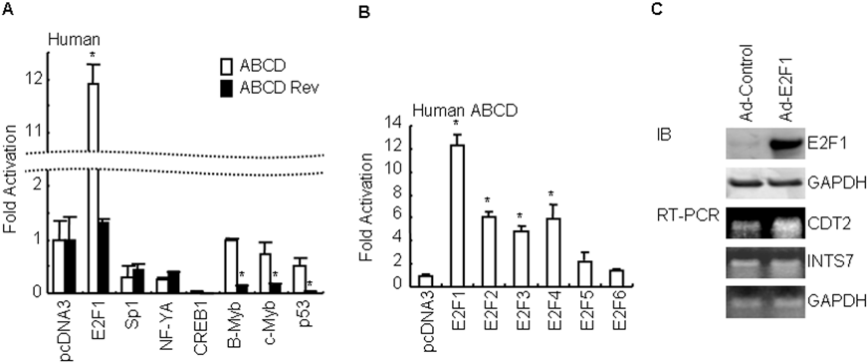
Transcription factors regulating human CDT2-INTS7 intergenic region. (A) Luciferase expression of pGL3-ABCD and pGL3-ABCD Rev constructs co-transfected with indicated expression vectors in A549 cells are shown as fold induction with respect to the pGL3-Basic vector together with pcDNA3 as 1. The values reported for transfection experiments are the means±standard deviation (n = 3; asterisk, *P*<0.05 for ABCD versus ABCD Rev). (B) Luciferase expression of pGL3-ABCD co-transfected with indicated E2F members in cells are shown as fold induction with respect to the pGL3-Basic vector together with pcDNA3 as 1. The values reported for transfection experiments are the means±standard deviation (n = 3; asterisk, *P*<0.05 for pcDNA3 versus E2Fs). (C) Overexpression of E2F1 in A549 cells by adenovirus transfer resulted in the upregulation of CDT2 but not INTS7 mRNA. Western blot analysis of E2F1 and GAPDH in A549 cells infected with Ad-Control or Ad-E2F1. GAPDH was detected as a control. RT-PCR analysis of CDT2 and INTS7 mRNA expression levels are shown with or without E2F1 overexpression. GAPDH was detected as a control. RT-PCR products were derived from amplifications in the log range.

To check the possibility that E2F1 might regulate the transcription in the INTS7 orientation, or which orientation of intergenic region might be regulated by other transcription factors, we used pGL3-ABCD and pGL3-ABCD Rev and co-expressed them with expression vectors for E2F1, Sp1, NF-YA, CREB1, B-Myb, c-Myb, or p53. As shown in Fig. 5A, E2F1 increased the promoter activity of pGL3-ABCD but not pGL3-ABCD Rev, indicating that E2F1 exclusively up-regulated promoter activity in the CDT2 direction and was not responsible for INTS7 expression through the intergenic region. Sp1, NF-YA, and CREB1 diminished both the pGL3-ABCD and pGL3-ABCD Rev luciferase reporter activity. Whereas B-Myb, c-Myb, and p53 had no effect on the luciferase activity from pGL3-ABCD, they caused a significant decrease in the promoter activity of pGL3-ABCD Rev. To address which E2F members were crucial for CDT2 expression, pGL3-ABCD was co-transfected with E2F family members. Our data indicated that E2F1∼E2F4 but not E2F5 and E2F6 could regulate CDT2 transcription (Fig. 5B). Taken together, some regulatory elements within CDT2-INTS7 intergenic region could asymmetrically regulate the transcription of both genes.

To further address whether CDT2 was regulated by endogenous E2Fs in living cells, we examined the CDT2 mRNA level by RT-PCR under the condition of adenovirus-mediated E2F1 overexpression. Western blot analysis confirmed the E2F1 overexpression in the Ad-E2F1 infected cells, whereas the Ad-Control infected cells expressed a little E2F1 protein (Fig. 5C). On the other hand, the GAPDH protein level was not affected in any of the infected cells. After 24 hours virus infection, RNA was recovered and processed for RT-PCR analysis. As shown in Fig. 5C, Overexpression of E2F1 resulted in the upregulation of CDT2, but not INTS7 and GAPDH, mRNA.

### Expression of the CDT2 and INTS7 between human and rodent is regulated independently

**Figure 6:**
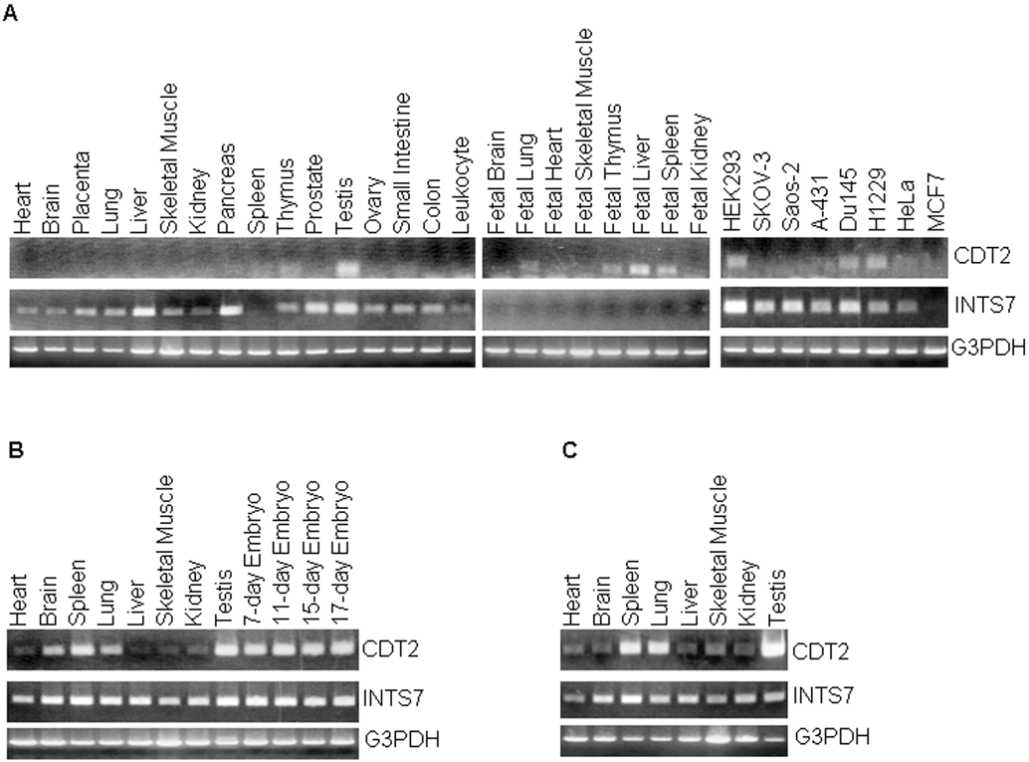
RT-PCR analysis of the CDT2 and INTS7 genes in various human (A), mouse (B), and rat (C) tissues and cell lines. The lower panels in each part show the G3PDH bands of the ethidium bromide-stained gels as a control. The source of the cDNA is indicated at the top. RT-PCR products were derived from amplifications in the log range.

The expression of the CDT2 and INTS7 in human and rodent tissues has not so far been investigated in detail. We investigated the expression of both genes through RT-PCR of the cDNA prepared from various human tissues and cell lines. As shown in Fig. 6A, human CDT2 was predominantly expressed in the testis, a tissue containing actively dividing somatic and germ cells, and slightly in the thymus. CDT2 transcripts were either undetectable or present at very low levels in most other adult human tissues. In contrast, human INTS7 was highly expressed in the liver and pancreas and modestly in all adult tissues examined except for the spleen. In case of fetal tissues, human CDT2 was clearly detected in the fetal liver, spleen, thymus, but only slightly in the lung, and no detectable expression in brain, heart, skeletal muscle, and kidney. Human INTS7 expression was not detected in all fetal tissues examined (Fig. 6A). While CDT2 was moderately expressed in HEK293, Du145, and H1229, expression of INTS7 was strongly detected in all the cell lines examined except for MCF7 (Fig. 6A). G3PDH expression was equally detected in all samples. The distinct expression pattern of human CDT2 and human INTS7 indicated that the expression of both genes was regulated independently.

The expression of CDT2 and INTS7 was further analyzed in rodent tissues. In mouse and rat tissues, the expression pattern of CDT2 was well conserved. Namely, CDT2 was highly expressed in mouse/rat testis, spleen, and lung tissues, and modestly expressed exclusively in mouse brain, and a weak signal was observed for the remaining mouse/rat tissues (Fig. 6B and C). Expression of CDT2 was highly detected in 7, 11, 15, and 17-day mouse embryotic tissue (Fig. 6B). INTS7 was constantly detected in all the rodent samples examined, as was the case for G3PDH (Fig. 6B and C). Taken together, both genes were expressed differentially between humans and rodents.

## Discussion

In the present study we found that the region shared by the two genes could possibly regulate bi-directional transcription, and that E2Fs play a critical role solely in the regulation of CDT2. In addition, we were able to show that the tissue and developmental expression patterns differed between CDT2 and INTS7, and also differed between human and rodent. For example, human CDT2 is predominantly expressed in the testis, while human INTS7 is ubiquitously expressed in all human tissues examined except for the spleen. On the other hand, rodent CDT2 is highly expressed in the testis, spleen, and lung and rodent INTS7 is expressed also in the spleen. Our data provided the first evidence that two genes, involved in DNA replication and transcription, respectively, could share a bidirectional promoter but have different regulatory mechanism.

Recent genome-wide studies have revealed that the genetic order in eukaryotic genomes is not completely random, but that genes with comparable and/or coordinated expression tend to be clustered together [7], [8]. Many genes are unexpectedly coupled by shared transcribed regions in antisense orientation by bidirectional promoters [9], [10]. The linkage of two genes by bidirectional promoters has been shown to facilitate control of functionally related genes [11], [12]. More examples still need to be added by individual experiments to expand the knowledge about head-to-head gene pairs to support the computational concepts deduced from the genome-wide approach. On the basis of this, the CDT2 and INTS7 genes provide a unique example considering that the two genes contribute to a different phenotype, namely, DNA replication and transcriptional regulation, respectively, though both phenotypes are characterized as part of the fundamental process of life. In the present study, the promoter activity was analyzed in both directions using expression constructs with luciferase as the reporter gene in transient transfection assays. Expression levels were comparable in both directions. The observation that CDT2 and INTS7 share a bidirectional promoter and that this architecture is conserved along evolution is very intriguing given that both genes are differentially regulated by transcription factors.

By assessing the nucleotide level identity for coding region, human CDT2 and INTS7 show 87 and 86% identity with rodents (mouse and rat) counterparts, respectively. This raises the possibility that highly conserved proteins between human and rodents being similar, the tissue distribution could be the same. Nevertheless, both genes were expressed differentially between humans and rodents. Moreover, the mRNA expression patterns of CDT2 and INTS7 in multiple tissues were inconsistent. Although the core promoter regions of CDT2 and INTS7 were highly conserved between human and rodent, species-specific enhancer(s) and upstream or downstream regulatory elements of the small intergenic fragment might be crucial for the expression pattern in tissues. Our present data suggested that unidirectional regulation could be achieved by transcriptional factors, at least E2Fs, through the highly conserved intergenic region of CDT2 and INTS7.

Other than the unique role of CDT2 as an essential component of the Cul4-DDB1 complex that controled CDT1 levels, CDT2 has been reportedly necessary for the early G2/M checkpoint to promote genomic stability in zebra fish [13]. In addition, CDT2 overexpression is known to be associated with the enhanced metastatic potential of hepatocellular carcinoma [14]. We revealed that E2Fs specifically up-regulate CDT2 transcription. Accumulating bodies of evidence suggest that E2F1 controls DNA replication, DNA repair, apoptosis, development, and is also involved in the regulation of the positive progression of tumors [15]. The E2F-CDT2 axis might therefore be a promising molecular clue to elucidate the etiology of carcinogenesis.

In conclusion, we present evidence that CDT2 and INTS7 may well be tightly linked by a bidirectional promoter in an evolutionary conserved manner. Within a short intergenic region, E2Fs could up-regulate gene expression in the direction of the CDT2 gene and B-Myb, c-Myb, and p53 could downregulate gene expression in the direction of the INTS7 gene. The tissue distribution of mRNA for CDT2 and INTS7 was inconsistent with each other. Moreover, the presence of similarities between mouse and rat tissue mRNAs were abundant, but these patterns were quite different from the results obtained from human tissues. These findings add a unique example and should help researchers understand the mechanistic insights into the regulation of gene expression through an evolutionary conserved intergenic region of the mammalian genome.

## Materials and Methods

### Bioinformatic analyses

CLUSTAL W (1.83) multiple sequence alignment was performed (http://align.genome.jp/sit-bin/clustalw). Prediction of the putative transcriptional factor binding sites was performed using Transfac software. Genomic sequences used for these analyses were human (NT_021877.18), chimpanzee (NW_001229613.1), canine (NW_876323.1), feline (AANG01612365.1), bovine (NW_001493454.1), mouse (NT_039189.6), and rat (NW_047402.1). cDNA sequences used were human (CDT2, NM_016448; INTS7, NM_015434), canine (CDT2, XM_547399; INTS7, XM_547398), and mouse (CDT2, NM_029766; INTS7, NM_178632).

### Construction of reporter gene plasmids

The CDT2-INTS7 intergenic region was PCR-amplified from human, mouse (Promega, Madison, WI), and canine (extracted from boxer) genomic DNA by using a forward primer 5′-GGGGTACCGTTTGACGCCATGACCCG-3′ (human pGL3-ABCD, mouse, canine, and also used for human pGL3-ABC, -AB, and -ABSp1) and a reverse primer 5′-GAAGATCTGCCTCCAACTCCCGCCACT-3′ (human pGL3-ABCD, mouse, canine, and also used for human pGL3-BCD and -CD). Deletion and inversion constructs were generated by PCR amplification of promoter fragments by using as a template the reporter plasmids pGL3-ABCD. The primers used were as follows; 5′-GAAGATCTCCTGGAGTCCAAGAGCTCCT-3′ (reverse primer for pGL3-AB), 5′-GGGGTACCTTCCCGCGGCTGGGAGAAAA-3′ (forward primer for pGL3-CD), 5′-GGGGTACCGACCCGAATAGTTACTCGAC-3′ (forward primer for pGL3-B and -BCD), 5′-GAAGATCTCTGACGTCACGCTCTCTGAT-3′ (reverse primer for pGL3-B and -ABSp1), 5′-GAAGATCTTCCGCGCCAAACTGACGTCA-3′ (reverse primer for pGL3-ABC), 5′-GGGGTACCGCCTCCAACTCCCGCCACT-3′ (forward primer for pGL3-ABCD Rev), 5′-GAAGATCTGTTTGACGCCATGACCCG-3′ (reverse primer for pGL3-ABCD Rev), 5′-GGGGTACCCCTGGAGTCCAAGAGCTCCT-3′ (forward primer for pGL3-AB Rev), 5′-GAAGATCTTTCCCGCGGCTGGGAGAAAA-3′ (reverse primer for pGL3-CD Rev). All the primer sequences included either KpnI or BglII restriction sites. After enzyme digestion, the fragment was cloned into the KpnI and BglII site upstream of the firefly luciferase reporter gene in the pGL3-Basic vector (Promega).

pcDNA3 and pcDNA3-E2F1∼E2F6, -Sp1, NF-YA, and p53 plasmids were as described previously [16]–[18]. CREB1 cDNA (NM_004379) was amplified with primers 5′-CGGGATCCGCCGCCATGACCATGGAATCTGGA-3′ including BamHI site and 5′-CCCAAGCTTATCTGATTTGTGGCAGTA-3′ including HindIII site, and cloned into the BamHI and HindIII sites of pCMV-Tag4A (Stratagene, La Jolla, CA). The mouse B-Myb and human c-Myb expression plasmids were provided through the generosity of Dr. Roger J. Watson (Imperial College London) and Dr. Bruno Calabretta (Thomas Jefferson University), respectively.

### Luciferase assay of promoter analysis

A549 cells were cultured in Earle's modified Eagle's medium (Invitrogen, Carlsbad, CA) containing 10% fetal bovine serum and penicillin/streptomycin (Invitrogen). The cells were cultured in a water-humidified incubator at 37°C in 5% CO_2_/95% air. A549 cells (3×10^4^) were transferred into 24-well plates with 500 µl of regular growth medium/well the day before transfection. Transfections were performed with the Fugene6 reagent as recommended by the manufacturer (Roche, Basel, Switzerland) with a mixture containing 0.2 µg of each reporter plasmid and 0.6 ng of pRL-TK (Promega), a plasmid that contains the *Renilla* luciferase gene under the cytomegalovirus promoter and is utilized as an internal control to normalize the effects of the transfection efficiency. Cells were lysed 24 hours after transfection by applying 100 µl Passive Lysis Buffer of the Dual Luciferase Reporter Assay Kit (Promega) into each well of the 24-well plate. Five microliters of cell lysate was used for the luciferase reporter assay with the same kit according to the manufacturer's protocol. Light intensity was quantified in a luminescence microplate reader (Wallac 1420 ARVOsx Multilabel Counter; PerkinElmer, Waltham, MA). The luciferase activity of the reporter plasmids was normalized to the *Renilla* luciferase activity. Each transfection experiment was carried out at least three times.

### RT-PCR analyses

The PCR was carried out in 25 µl of a mix consisting of 1× buffer, 200 µM dNTPs, 400 nM primers, 1 mM MgSO_4_, and 1 unit of KOD plus DNA polymerase (Toyobo, Osaka, Japan). As a template, 2.5 µl of cDNA purchased from Clontech (Mountain View, CA) was used (Human I, II, Fetal, Cell Line, Mouse I, and Rat I). The reaction consisted of 30 cycles (25 cycles for G3PDH and 35 cycles for rat CDT2), each cycle consisting of a denaturation step (94°C for 15 sec), an annealing step (60°C for 30 sec), and an extension step (68°C for 30 sec). PCR conditions for rat INTS7 was 30 cycles, each cycle consisting of a denaturation step (94°C for 15 sec) and an annealing/extension step (68°C for 30 sec). The first cycle was preceded by a denaturation step of 3 min at 94°C and the last one was followed by an extension step of 3 min at 68°C. The resulting PCR fragments were CDT2 (human, 304 bp; mouse, 480; rat, 500 bp), INTS7 (human, 380 bp; mouse, 510 bp; rat, 439 bp), and G3PDH (human, mouse, and rat, 983 bp). The primer sequences were as follows; human CDT2, 5′-CCATATCCCTGAGGACTGTGT-3′ and 5′-TTCCCAAAGCCCAACAGTCA-3; human INTS7, 5′-AGACTGGTCCCAGAACTACC-3′ and 5′-CTTGATCTCCTCGTGAGCCG; mouse CDT2, 5′-TCTCTGGGGGCTAGCTAAAC-3 and 5′-TCAGCTCAAGGTCACACGGC; mouse INTS7, 5′-TGCTGCATTGGCACCTCTTA -3′ and 5′-TTAGCAGCCCACTGCACCCA; rat CDT2, 5′-AAAGCCGGCCCAGTATCGGC-3′ and 5′-AGACTCTCCACTTGGCCGTC; rat INTS7, 5′-GCGTTGTTCAGCACGGGTCT-3′ and 5′-TGCAGTGTGGTAGCCGCATG.

### Adenovirus infection and Western blotting

Preparation of adenoviral vectors, virus infection, and Western blotting were performed in a basically similar manner as previously described [19], [20].
